# Exploring the villus 

**Published:** 2018

**Authors:** Arzu Ensari, Michael N Marsh

**Affiliations:** 1 *Department of Pathology, Ankara University Medical School, Sihhiye 06100, Ankara, Turkey*; 2 *Luton & Dunstable University Hospitals NHS Trust, Wolfson College, University of Oxford, UK *

**Keywords:** Small intestinal villus, Permeability, Epithelium

## Abstract

The small intestinal villus and its associated epithelium includes enterocytes as the main cell type and differentiated goblet and argentaffin cells, while the invaginated crypt epithelium is the site of cell division and hence the origin of all epithelial components. Enterocytes form a cohesive monolayer which acts both as a permeability barrier between lumen and the interior, and an important gateway for nutrient digestion, absorption and transport. Differentiation and polarisation of enterocytes depends on cytoskeletal proteins that control cell shape and maintain functionally specialised membrane domains; extracellular matrix (ECM) receptors; channels and transporters regulating ion/solute transfer across the cell. The mesenchymally-derived basement membrane dynamically controls morphogenesis, cell differentiation and polarity, while also providing the structural basis for villi, crypts and the microvasculature of the lamina propria so that tissue morphology, crucially, is preserved in the absence of epithelium. Mucosal re-organisation requires immense cooperation between all elements within the lamina, including marked revisions of the microvasculature and extensive alterations to all basement membranes providing support for endodermal and mesenchymal components. In this context, subepithelial myofibroblasts fulfil important regulatory activities in terms of tissue morphogenesis; remodelling; control of epithelial cell development, polarity and functional attributes; and an intimate involvement in repair, inflammation and fibrosis.

This paper reviews the main structural and functional aspects of the villus, including the epithelium and its outer glycocalyx and microvillous border; and subjacent to the epithelium, the basement membrane with its attached web of myo-fibroblasts together with the lamina propria core of the villi, and its microvasculature and lacteals. Finally, some comments on the rapidity with which the overall structure of the villi changes in their response to both external, and internal, influences.

## Introduction–Why "Villus"?

 Although the Latin "villus" refers to the "shaggy haired" nature of animals coats, Gabriele Falloppio (1523-1562), of tubal fame, first used the word "villi" in his 16th Century text "Observationes Anatomicae" (1561). However, his description derived from the feel and texture of velvet (1): any connection with the Latin is thus obscure.

 During the 17-18th Centuries, microscopic anatomy did not exist, so that intestinal "villi" were deemed analogous to dermal papillae (rete pegs), since the overlying cellular basis of either dermis and epithelium – yet to be discovered - was simply regarded as an amorphous, gelatinous surface coating. One can therefore understand why X. Bichat (1771-1802) came to employ the term "mucous membrane" for the first time in his "Traite des Membranes" (2) – although goblet cells had yet to be recognised. Thus intestinal "villi", at first, were only perceived in terms of their central cores, being seen as small, upwardly-projecting structures in wet preparations, their vessels defined through injections of red and green wax into mucosal arteries and veins.

 The widespread use of microscopes during the 19th Century gave rise to the earliest descriptions of the cellular nature of the so-called "epithelium" (coined from the Greek ἐpi = upon, and θhlή = nipple). Two almost simultaneous sources for the discovery of epithelial cells came from F.G.J. Henle (of the renal tubular loop) in Germany (1837) (3), and W. Bowman (of renal capsular fame) at King's College, London (4). However, Bowman's predecessor, Robert Todd, revised Henle's use of "prismatic" to "columnar" in describing individual cells.

 A modern account of the villus and its associated epithelium includes the differentiated goblet and argentaffin cells, while the invaginated crypt epithelium is the site of cell division and hence the origins of all epithelial components. This paper reviews the main structural and functional aspects of the villus, including the epithelium and its outer glycocalyx and microvillous border; and subjacent to the epithelium, the basement membrane with its attached web of myo-fibroblasts and cognate cells – pericytes, smooth muscle, fibroblasts and muscularis mucosae, and outer cells of Cajal. Here we encounter the lesser explored lamina propria core of the villi, which includes the microvasculature and lacteals. Finally, some comments on the rapidity with which the overall structure of the villi changes in their response to both external, and internal, influences.

**The epithelium and brush border – the ins and outs**

 The intestinal tract is lined by a single layer of columnar epithelium originating from multipotent stem cells at the base of each crypt, giving rise (5) to four major types of epithelial cells: (i) absorptive enterocytes comprising >80% of all small intestinal epithelial cells; (ii) goblet cells producing various mucins and trefoil peptides needed for epithelial growth and repair; (iii) entero-endocrine cells which export peptide hormones; and (iv) Paneth cells which secrete antimicrobial cryptidins or defensins, digestive enzymes, and growth factors. Following their differentiation, enterocytes, goblet and entero-endocrine cells migrate upwards thus to be exfoliated (from presumptive "extrusion zones") (6) at the villous tips after approximately five days (7). Transfection of basal crypt cells with marker proteins permits examination of the successive changes occurring in enterocytes as they leave the crypts (8). Indeed, distinctive switches in gene expression patterns during contact with neighbouring cells casues down-regulation of differentiation signals in favour of those now necessary for specialised functions as polarised cells, especially at the brush border zone (9,10 ).

 Complex signaling pathways implicated in the regulation of specific differentiation of the cells in the intestine include Wnt -β-catenin-TCF, Notch and its downstream effectors - HES1 and Math1, BMP-TGF-β-SMAD, and hedgehog (Hh). A number of transcription factors, many of which are downstream targets of these signaling pathways, including cdx-1 and cdx-2; kruppel-like factor; GATA4, 5 and 6 together with several forkhead family members, E-cadherin-mediated cell-cell and integrin-mediated cell-matrix adhesion; chemotactic gradients; extracellular matrix and mesenchymal components; and a range of cytokines, hormones and growth factors, have each been implicated in the regulation of intestinal cell maturation. This is too expansivea field for detailed discussion here.

 As newly-formed enterocytes migrate from the crypts, they develop the apical "brush border" comprising microvilli approximately 1μM in length and 0.1μM in diameter. There are approximately 3,6000 (±450) microvilli per cell (Marsh, unpublished) thereby increasing the surface area by ~10-20 fold (11). The resulting increased 'reserve' of surface membrane allows additional specialised functions permitting membrane-associated macromolecular digeston and absorption (12,13), and facilitating defences at this important host-environmental interface (14). 

**Figure 1:**
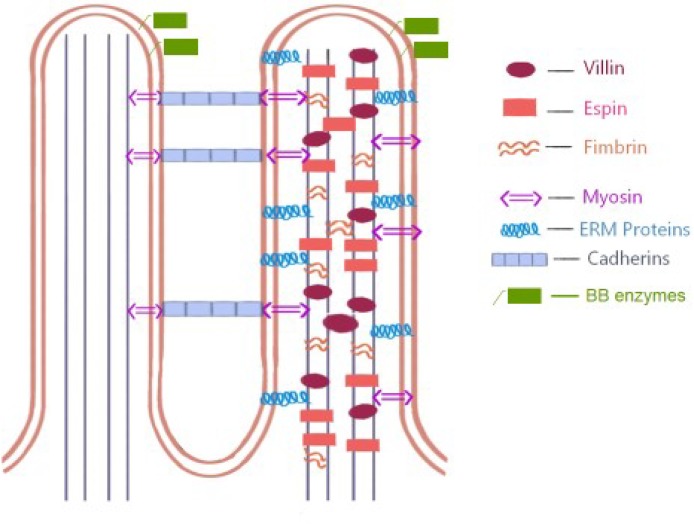
This diagram represents the molecular structure of the BB. Actin filaments within each microvillus are bundled by villin, espin, and fimbrin which also serve to stabilize the actin core. Molecules such as unconventional myosins and ERM (ezrin, radixin, moesin) family proteins cross-link the plasma membrane to the underlying actin cytoskeleton while extracellular adhesion molecules such as cadherin family members—protocadherin-24 (PCDH24) and mucin-like protocadherin (MLPCDH) mediate intermicrovillar adhesion during brush border assembly. Between the external and internal surfaces of the microvillus membrane brush border enzymes are located. Adapted from Crawley et al (12).

 Together, enterocytes form a cohesive monolayer which acts as a permeability barrier between lumen and the interior, and as an important gateway for nutrient digestion, absorption and transport (15). Differentiation and polarisation of enterocytes depends on cytoskeletal proteins (16,17) that control cell shape and maintain functionally specialised membrane domains; extracellular matrix (ECM) receptors; channels and transporters regulating ion/solute transfer across the cell (Figure 1). As already hinted, the microvilli together with their enzymes and transporter proteins represent the most important functional differentiation within the villus for digestion/absorption of carbohydrate, proteins, lipids, minerals and vitamins. Subsequent processing within the enterocyte requires the help of secretory and sorting pathways.

**Figure 2:**
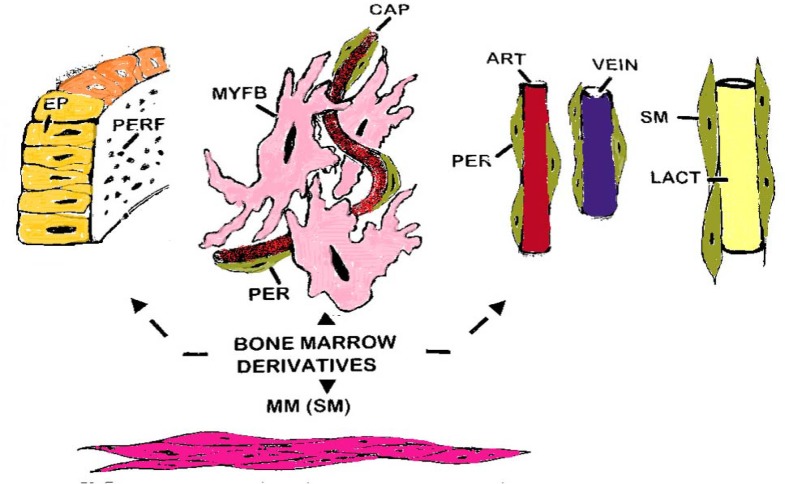
This diagram represents the types of bone-marrow cell derivatives operative within the lamina propria. They include (in cerise) the subepithelial myofibroblast system (MYF); pericytes (green) supporting the subepithelial capillaries and main vasculature of the villi (artery, red: vein, blue); the lacteal (L) supported by smooth muscle cells (SM) and (purple) the muscularis mucosae (MM). The basement membrane (green) is perforated (but artefactually so during processing for microscopy), comprising glycoglucosamines and fibres (such as collagen IV, tenascin, elastin, etc) which are all largely derivative of the mesenchymal cell populations illustrated

**The basement membrane – potentiating potential**

 Histologically, basement membranes appear as insignificant thin strips of homogeneous, amorphous pink material lying between epithelium and subjacent mesenchymal elements, such as myofibroblasts. Many years ago, this region was shown to be periodic acid- Schiff positive and hence rich in glycoprotein(s), while electron microscopy revealed the presence of fibrillar material.

 Basement membranes are ubiquitous throughout the body, containing laminin-1, fibronectin, type IV collagen, entactin (nidogen), together with perlecan (18) as the major component of heparan sulphate proteoglycans (HSPG) (19). Laminin and collagen IV are mainly synthesised by mesenchyme, while HSPG derives from the basal epithelium, as also entactin (nidogen) (20). However, fully-formed and functional basement membrane depends critically on mesenchymal derivatives. Moreover, laminin A chain synthesis is accelerated, and selectively, as the foetal intestine matures towards the end of gestation with the onset of epithelial cell differentiations (21,22). There is also evidence of spatial orientation of HSPG and laminin chains more-or-less orthogonally across the basement membrane, thus not only strengthening the intermolecular cross-binding of these cruciate molecules (23), but also providing specific anchorage between them and overlying cell membranes. Their molecular dimensions indicate their capability of spanning the entire thickness of basal laminae (24,25 ).

 Yet despite these recent insights, several problems remains (26): are basement membranes homogeneous throughout, both along the entire intestinal tract and at all levels between the crypts and villous tips? what relationship exists between the upward migration of the epithelium and possible molecular changes in the membrane at the so-called extrusion zones at tips of villi? and are cells only extruded from the upper reaches of the villi given, for example, the vast excess of crypts in some species? (27). These anatomical variants both in crypt ratios to villi and the structural variations between pencil-shaped and leaf-shaped villi, call into question the often-assumed existence of extrusion zones located (only) at villous tips. Very careful studies on resected pieces of full thickness bowel have been disappointing in revealing site(s) of desquamation (28), while the locus of the increased rate of enterocyte loss in flat coeliac mucosae, for example, has never been precisely defined. Indeed, the concept of "extrusion zones" at villous tips seems rather more a mythology than reality, perhaps. Rather, it is more likely that cells are removed at all points along the villous epithelium (29) even from the inter-villous regions of the mucosa (30) and may be the reason why desquamated cells are very difficult to observe, especially when only thin histological sections are employed for identification.

 Another problem is how the epithelium, collectively, moves along the basement membrane. Earlier studies suggested that epithelium and subjacent myofibroblasts move upwards in a cohesive manner (31,32) but that may not be so (33): indeed, we have very little knowledge about the turnover of these cells and their means of replacement. We must also remember that due to villous tip narrowing, the changing apical geometry demands considerable shedding if the cells remaining are to retain contact with the basement membrane. That difficulty remains to be elucidated.

 The appearance of the basement membrane as a continuous sheet is probably artefactual on account of the coagulation of its glycoprotein elements during fixation and processing: but scanning EM has drawn attention to "holes" (~0.5-5μM diameter) disrupting its continuity (34,35). It hardly seems likely that this membrane arises de novo as a perforated sieve. Rather, these perforations must therefore reflect those items, like migratory cells, basally-extruded enterocyte projections, together with inert material, which pass through it. It was shown in earlier studies that the membrane could be breached, albeit with some apparent mechanical difficulty. In those studies of inflammatory exudates, Sir Howard Florey in Oxford noted membrane bulging in the presence of emigrating inflammatory cells (36,37), thus rejecting ideas of being 'softened up'. That is presumably incorrect, since malignant cells when breaking out of the conformity of a regular epithelium do secrete enzymes, most crucially against collagen IV (38,39). This information, additionally, corroborates the specific mechanical contribution of this protein in maintaining structural integrity of basement membranes. Conversely, it is unlikely that transmigrating lymphocytes have such aggressive properties. Neither would the passive (?) movement of chylomicrons (0.5-1.0nm diameter) require such behaviour, despite their obvious movement through the membrane (40,41). A more likely explanation for these breaches probably rests in the physic-chemical make-up of the basement membrane itself which momentarily dissolves under local forces (rather like thixotropic "non-drip" paint which liquefies only under pressure from the paintbrush) (42).

 Another intriguing relevant perspective, neither fully explored nor resolved, involves the presence of epithelial cell pseudopodia traversing the membrane to contact mesenchymal cells. It is probable that the marked increase in number and size of projections during end-gestational development (43) may well correlate with the activity of mesenchyme in promoting epithelial maturation and polarity, and hence functional capacity and potential (9,11,44). That momentary cross-talk between epithelium and mesenchyme, unfortunately, has neither been entirely corroborated nor defined.

 To what extent, therefore, do messages from the mesenchyme control and direct epithelium? We have seen that a mesenchymally-derived basement membrane dynamically controls morphogenesis, cell differentiation and polarity, while also providing the structural basis for villi, crypts and the microvasculature of the lamina propria, so that tissue morphology is preserved in the absence of epithelium. Now, in terms of mucosal (coeliac) "flattening", the frequently asserted presumption that loss of epithelium explains altered surface topology, especially by immunologists (45,46) whose conclusion, that IEL primed for enterocyte cytolysis provides the answer, is hardly sufficient. 

 But as we have argued elsewhere (47,48) such views are highly suspect since mucosal re-organisation requires immense cooperation between all elements within the lamina, including marked revisions of the microvasculature and extensive alterations to all basement membranes offering support to endodermal and mesenchymal components. Hence, we need to be very sure, even in these immune-driven situations, whether the force for change comes totally, or partially, from controlling influences elsewhere, or more specifically perhaps, from within the mesenchyme itself. Indeed, the mesenchyme may be the originating force which controls tissue shape and integrity to a far greater extent than has ever been realised hitherto, even to contributing to the innate immune response of tissues: a point recently articulated by others (49,50,51).

**The lamina propria & the myofibroblast subepithelial cell system**

In stark contrast to other studies on mucosal morphology and the villous epithelium, detailed analyses of the lamina propria have not been so prominent, either in regard to normal functioning or to pathological processes. Although the lamina propria contains a variety of haemopoietically-derived infiltrating cells (eosinophils, basophils, neutrophils, lymphocytes), we know little about its basic content of residual structural cells, their associated macromolecular repertoire (matrix proteoglycans), and how these are altered by specific disease processes. Histologically, the lamina is difficult to analyse, rather more being regarded simply as the region lying between the crypts and forming the internal villous core. 

 However, the lamina is bounded by a system of myofibroblasts which is intimately applied to the under-surface of the basement membrane, and directly connected with the subepithelial capillary vascular network of pericytes (52,53,54,55,56). In addition to various fibrous elements (including collagen IV, tenascin, desmin, entactin, laminins), the "ground substance" comprises a gel-like mixture of glycoaminoglycans of which hyaluronic acid is a prominent component. The degree of hydration of this matrix is dependent on the balance of absorbed fluid, secretions, and rate of removal through the microvasculature and lymphatics. Each villus is supplied by a central arteriole which branches into capillaries at its tip into a local tuft. This begins draining into a venule which takes origin from about the upper one-third of the villus. Below this tuft, the lower capillaries drain vertically downwards, subjacent to the basement membrane and fibroblast sheath, where they join vessels associated with the crypt mouths (57, 58): the dual structure of the subepithelial capillary sheath in human intestine should be noted. The central lacteal is clothed in smooth muscle cells in its upper part before its division into smaller tributaries, the latter only being supported by pericytes (also now considered to be part of the myofibroblastic system) (Figure 2).

 In view of recent important advances over the last few decades, attention should now be focussed on the myofibroblast system throughout the lamina propria and the different phenotypes emerging from within that system (59,60,61). It comprises a ubiquitous population of cells (62) that is usually α-SMActin + (smooth muscle), tenascin-C+ and desmin+, but in intense inflammation, as with Crohn's or IBD disease (63), they are prone to lose these markers when de-differentiating into fibroblasts which may also therefore be the source of intestinal fibrosis and stricture formation. 

 Their precursors could all derive from the bone marrow (64). This was demonstrated in an ingenious use of intestinal biopsy material from female patients thought to be developing GVHD after receipt of whole blood transfusions from male donors. Using the Y chromosome as evidence, they detected α-SMA+ donor cells within the pericryptal myofibroblast sheath of three biopsies. Therefore, these migrating cells probably arose from stromal cells, and possibly from among circulating "fibrocytes" (65) within the bloodstream. Interestingly, although not formally interrogated, these donor cells appeared to reach onto the villi, so the possibility of their migratory potential has not yet been entirely ruled out.

 Subepithelial myofibroblasts, histologically, reveal a smooth muscle appearance but which variously (according to organ/tissue type) fulfil important regulatory activity in terms of tissue morphogenesis; remodelling (following injury); control of epithelial cell development, polarity and functional attributes; and an intimate involvement in repair, inflammation and fibrosis. In addition to the subepithelial fibroblast network, this group of cells also provides the pericytes in support of the microcirculation, as well as the vertically-aligned smooth muscle supporting the lacteal system within each villus. Their contractile properties are thus probably responsible for the contraction of individual villi, as presumably indicated by their 'fir-tree' profiles seen histologically, and in the horizontally-disposed folds revealed by scanning EM, thereby encouraging effective fluid transfer, and probably also aiding loss of cells from the epithelial surfaces.

**Evaluating the villus – from normal to abnormal**

 This brief review emphasises the complexity of the structural, cellular, non-cellular, and gene-based aspects of villus-orientated biology, exemplifying, over the last 50 years, the vast expansion in knowledge applicable to its various parts. In addition, the villus is subject to varied influences, as typified by responses to gluten ingestion in genetically-predisposed individuals (66,67); high bacterial intestinal loads resulting in the host-directed syndrome of tropical ("sprue") enteropathies (68,69); or parasites - especially Giardia species (70), all of which evoke profound changes in villous morphology. While some of these changes are minimal, others result in significant re-modelling of the mucosa architecture: the spectrum of immunopathologic changes is tabulated for comparison (Table 1). 

 Much of the work done on these conditions, although principally from a clinico-pathological, diagnostic viewpoint, has centred specifically on the epithelium and its lymphocytic infiltrations; the shape of the villi; on the nature of its severest changes ("flattening"); with very scant attention being given to the lamina propria. These progressive changes (related in the main to gluten-induced hyper-sensitivity reactions) and the relevant computer-aided morphometric data have recently been schematically illustrated (48) thereby indicating both the time-frame, and key locations within the mucous membrane at which these changes are progressively initiated.

**Table 1:** Some prominent pathologies leading to villus deformities

	«Normal»/ Preinfiltrative Marsh 0	Infiltrative Marsh I	Infiltrative/ Hyperplastic Marsh II	«Flat»/ Mosaic Marsh III	«Flat»/ Unresponsive Marsh IV
Disorder					
Gluten hypersensitivity^a^	+	+	+	+	+
Tropical sprue^b^	+/-	+	+	+	-
Chronic diarrhoea/Marasmus^c^	+/-	+	+	+	-
Giardiasis/infections^d^	+	+	+	+	-
GVHD^e^	-	+	+	+	-
Food antigens^f^ Milk Egg Soya Chicken	- - - -	+ - + -	+ - + -	+ - + +	- - - -
Transport and enzyme disorders^g^ Carbohydrate intolerance Abetalipoprotein-aemia Chylomicron retention disease	+ + +	- - -	- - -	- - -	- - -
Immunodeficiencies^h^ CVID	+	+	+/-	-	-
IBD^i^	+	+	+	-	-
Drugs (NSAIDs)^j^	+	+	+	+/-	-
Neonatal enteropathies^k^ Microvillus inclusion disease Tufting enteropathy Enteroendocrine cell dysgenesis	+ + +	+/- - -	+/- - -	+/- - -	- - -

(i) These gluten-induced and other hypersensitivity reactions lead to alterations in villous shape, ultimately being involved in the hyperplastic remodelling of mucosa into mosaic plateaux. Villi do not undergo *atrophy*, however, and neither is the mucosa subject to any *atrophic process*, since structural recovery ensues following use of a gluten-free diet:

(ii) The minimal change mucosal lesions (Marsh I and II) cannot be *non-specific*, because they represent specific host responses to identified inciting antigens:

(iii) The subdivision of Marsh III (a, b, c) has been shown in various independent studies to have no practical value: this misinterpretation results from the failure to recognise the elevated mosaic plateaux which amalgamate villi into these lozenge-shaped blocks of tissue. It follows [see Note (i]) that attempted sub-classification of the severe Marsh III lesion are futile, and would be better to be abandoned:

(iv) Immunodeficiency’s, Inflammatory bowel diseases (IBD), particularly Crohn’s disease and drugs may cause intraepithelial lymphocytosis similar to the Marsh I and II lesions while flat mucosa is only rarely encountered.

 Myofibroblasts, as we have shown, arise in the mucosa from circulating fibroblasts, and which in the presence of pro-inflammatory proteins synthesise extra-cellular matrix (ECM). If the latter is disorganised through tissue remodelling (as signally occurs in the evolution of the characteristic coeliac mucosa), fibroblasts acquire stress fibres, gradually being transformed into mature myofibroblasts expressing α-SMA. Myofibroblast differentiation requires three stimuli: a) TGF-β1); special ECM proteins like ED-A (a splice variant of fibronectin); and c) stress resulting from tissue remodelling (71,72). Despite that, and in contrast to pulmonary fibrosis, liver cirrhosis, or the chronic cicatrizing fibrosis of Crohn's disease, extensive fibrosis is not a major feature of celiac mucosae, the former possibly due to a breakdown in epithelial-to-mesenchymal (EMT) transitions (73).

 EMT interactions are essential in foetal intestinal development and later adult architecture, a process critically dependent on epithelial Hedgehog (Hh) in the formation of the lamina propria. Hh binds to Patched (Ptch) on target cell membranes (74, 75). Indeed in studies of mice programmed for chronically-reduced Hh signalling, the result was the development of diarrhoea, malabsorption, weight loss and malnutrition accompanied structurally by a reduced villous heights, crypt hypertrophy and inflammation in the lamina (76). In addition, there was loss of smooth muscle leading to failure of lacteal development, here analogous with intestinal lymphangiectasia.

 The purpose of this essay was not primarily to notice the acquired pathology of diseases affecting the villus. Nonetheless, these very brief remarks illustrate just how little is known about the remodelling of the mucosa in terms of genetic activation or deficiency, indicating the vast chasm which needs to be investigated, understood and further integrated into current understandings of mucosal disease. Their relevance to celiac disease is immediately apparent, and again with observations which distract strongly away from the idea that mere "atrophy" has anything to do with these profound tissue re-arrangements.

 However, in light of this review, it is clear that much more work needs to be done on small bowel enteropathies regarding changes in the lamina propria and the role taken by the syncytial subepithelial myofibroblast sheath. Mucosal re-modelling is not simply related to loss of epithelial cells, as often widely assumed. This, therefore, remains a challenge. It requires further investigation of the Hedgehog and Wnt series of genes (77) which exert such major influences on mucosal structure, development, maintenance, and pathology. It is inconceivable that this feature of the mucosa does not exert a prominent role in the immunopathogenic changes which have already been described: some encouraging starts have already been made (78) and which should stimulate further research in this area, thus bringing newer insights into how these changes are brought about – and, of course, reversed following treatments.

## Conflict of interests

The authors declare that they have no conflict of interest.
